# Dental Convention of Northern Ohio

**Published:** 1858-06

**Authors:** 


### DENTAL CONVENTION OF NORTHERN OHIO.

### MINUTES AND DISCUSSIONS.

The Dental Convention of Northern Ohio, held its second session in Tremont Hall, Cleveland, on Tuesday and Wednesday, the 4th and 5th of May, 1858.

Members present:

F. S. Slosson, B. Strickland, Henry Slosson, G. Langsdoff, E. G. Barger, B. A. Halliwell, B. F. Robinson, C. R. Butler, J. Koch, J. G. Moore, Cleveland; G. H. Perine, New York; C. V. Atkinson, Michigan; J. B. Irwin, G. Scott, Carrolton, O.; C. F. Ingersoll, Huron; W. B. Ingersoll, Oberlin; A. E. Lyman, Newton Falls; J. F. Siddall, Oberlin; L. C. Ingersoll, Keokuk, Iowa; Alfred Terry, Norwalk, O.; S. P. Huntington, Painesville; E. J. Way, Sandusky; B. T. Spelman, Ravenna; J. A. Robinson, Litchfield; J. E. Atkinson, Millersburg; W. E. Dunn, Delaware; S. D. Tuttle, Albany; Geo. Watt, Xenia; W. J. Guild, Cleveland.

### TUESDAY—MORNING SESSION.

The President, Dr. Slosson, took the chair, at half past ten, and called the meeting to order. The reading of the minutes of the last meeting was dispensed with. The Executive Committee was called on to report. Dr. Strickland, the only member of the committee present, reported the following order of business:

I. President's Address.

II. Election of Officers.

III. Discussion of the subjects reported in the circular of the committee, viz:

1. Under what circumstances is it NOT admissible to extract teeth?

2. Can teeth generally be preserved for a considerable length of time, after abscess has formed around the roots?

3. Is it judicius to use arsenic, or any other powerful agent, to destroy the sensibility of dentine?

4. What treatment will best preserve the vitality and health of exposed dental pulps?

5. What preparation of gold, and what mode of inserting it, will most certainly and permanently arrest the progress of decay in teeth ?

IV. Miscellaneous Business.

The report was accepted and adopted; but, as several members yet absent were expected on the forenoon trains, on motion of Dr. B. F. Robinson, the President's Address, and the election of officers were postponed till afternoon. On motion of Dr. W. H. Atkinson, it was agreed to spend the remainder of the morning session in miscellaneous business, without regard to programme.

Dr. G. H. PERINE, by request, made a few remarks on the condition and prospects of the profession in New York.

Dr. W. H. ATKINSON read a letter from J. B. Trembley, M. D., detailing an interesting case of facial paralysis, induced by dental disease. On motion of Dr. Perine, the letter was received and ordered to be embodied in the minutes. The letter is as follows :

*Gentlemen of the Northern Ohio Dental Convention*:—Not having the pleasure of meeting you in Convention as I anticipated, and through the request of our friend, Dr. W. H. Atkinson, I submit the following case that has fallen under my observation, which may not be void of interest to the gentlemen in attendance upon your convention.

The case was a child of nine years of age, had had violent pain in the first deciduous molar tooth, on the left side of the inferior maxillary, which inflamed, suppurated, and was removed. During this progress of pain and suppuration, a paralysis of the muscles on the left side of the face came on, giving the child a grotesque and clownish appearance, so much so that she would run and hide when strangers came into the house, for fear of being laughed at. The orbital, cronal, nasal, superior labial, and inferior labial groups of muscles on the left side, were wholly uncontrollable by volition. The skin was as sensitive as ever to all external impression over the muscles paralyzed. That the cause of the paralysis was depending upon the diseased tooth, there can be no doubt; but how it should produce such a peculiar paralytic effect upon the muscles that were effected in the little patient, would, perhaps, come more under the observations and speculations of the pathologist and physiologist, than that of the dentist. However, as the field of dentistry, for the few past years, has been greatly extended, and many diseases, both of the *face* and *alimentary canal* have a more intimate relation with the condition of the teeth than was formerly supposed. I presume that the above case referred to, with the following speculations or relations, may not be uninteresting, or fail to lead enquiring minds to a more close analysis of many diseases that are met with, both by the physician and dentist.

Here was a case which certainly arose from the irritation of an ulcerated tooth, acting upon the nerve leading to it; which irritation, extending from that, was reflected to the muscles above referred to. The question arises, how did this reflex action take place, to produce the phenomenon witnessed, without a more extensive paralysis than that which existed? The nerve immediately affected was the *inferior dental nerve*, a branch of the inferior maxillary, or third branch of the fifth pair. From this, anatomically considered, the irritation must have extended through the *mental foramen* externally, where it anastomoses with the branches of the facial nerve of the seventh pair. Also, up the inferior maxillary to the ganglion of *Casser;* then along the *superior maxillary* to the *infra orbital* nerve, where it emerges from the *infra orbital foramen* ; also, along the first, or ophthalmic to the frontal nerve, to the *supra orbital foramen.*

In this case the muscles were paralyzed only by those nerves that passed from these various *Foramina*, viz., stylomastoid, infra mental, infra orbital, and supra orbital; for within these foramina, the nerves appeared to perform their functions properly. Externally only, were their motive powers lost, the nerves of sensation were as acute as ever. Physiologists tell us that the *facial nerve* is an exclusive *motor*; but the above case leads to some doubt relative to their assertions, and leaves the physiology of the nerves open for further investigation, relative to its functions, distributions, and anastomosis.

Respectfully your co-laborer in the field of scientific and medical research.

J. B. Trembley, M. D.

Florence, May 3, 1858.

A member requested Dr. A., before taking his seat, to tell how he treats "tooth-ache."

Dr. A. said he would remove the cause. If it arise from congestion of the pulp, remove the pressure. If by uncapping, or removing the decay, it was not relieved, he would puncture it. But if the difficulty be with the periosteum, he would cut boldly through the gum, the process, and the periosteal coverings, to the end of the fang. It is in the congestive stage of periostitis, that pain is principally felt. It may look like barbarous practice, but it is the treatment. The congested periostisms cut as if highly elastic, like India Rubber. Another form of tooth-ache is pure neuralgia. In this he would endeavor to divert the nervous action, which can often be done by electricity, friction, anodynes, or other means. He uses creosote as a local application, both in congestion of the pulp and in periostitis.

Dr. STRICKLAND would suggest other treatment, if not more efficient, perhaps more acceptable to the patient; yet he was not to be understood as doubting the success of Dr. A's treatment. When a tooth was painful from death of the pulp, and consequent suppuration, he would open the cavity and remove the dead matter, then apply creosote; and, having removed the cause, he would expect relief. In swelling from periostitis, he would leech the gums. He said there was often a tendency to abscess around roots of teeth which have long been dead. He would expect less relief from treatment in the fang in such cases.

Dr. DECAMP was requested to give the history of one of his own teeth now under treatment, and stated that eleven years ago one of his cuspid teeth began to ache suddenly. On examination he found that the nerve was exposed. This was destroyed with arsenic; and the tooth was filled temporarily with tin foil, the canal not being filled. This filling, however, was allowed to remain till recently. There was always a little tenderness at the root; and about two weeks ago the pain became severe, and the filling was removed, followed by a free flow of blood from the canal. Bleeding had occasionally occurred to a slight extent since. Last evening, Dr. W. H. Atkinson opened the canal, and the pain was instantly relieved.

Dr. ATKINSON, by request, described his treatment of the tooth thus far. He enlarged the canal and made a free opening through the apex of the fang, washed out with water and tincture of arnica, then pumped creosote through the canal, followed by tincture of iodine.

Dr. STRICKLAND inquired if there was any danger of forcing air through the apex of the fang, as had been suggested by a recent writer? Dr. A. thought there was not.

Dr. SLOSSON reported a case of diseased alveolar process, with perforation of the antrum, through the socket of a tooth which had been previously extracted.

Dr. W. B. INGERSOLL reported a case of a child five years old, attacked with what was thought to be typhoid fever. The third day after the attack the teeth were all loose, so much so, that a few days later the child picked some of them out with its fingers. The case, as might be expected, proved fatal. He did not know what medical treatment the child received, but thought it had little or none till near its death.

On motion of Dr. B. F. Robinson, the Convention adjourned till 2, P. M.

FIRST DAY—AFTERNOON SESSION.

The President, before leaving the chair, read an address on "Professional Character," which was listened to with attention, and greeted with applause.

On motion, the thanks of the Convention were tendered to the President for his interesting address, and a copy was solicited for publication. (See page of present number.)

The election of officers resulted as follows: B. STRICKLAND, President; S. P. HUNTINGTON, Vice President; C. R. BUTLER, Recording Secretary; B. F. ROBINSON, Corresponding Secretary and Treasurer.

The following resolutions, offered by Dr. Perine, were unanimously adopted:

*Resolved,* That there be a time set apart for members to present or exhibit instruments, casts, cuts, or anything new or useful to the profession.

*Resolved,* That this Convention recommend the formation of local societies in all places where they may be sustained, for the purpose of regulating fees, for general improvement, and to promote good feeling and unity of action among the profession.

Dr. W. H. ATKINSON read a paper which was highly applauded, and for which the thanks of the Convention were unanimously given. (But we have not the paper.—W.)

The member of the Executive Committee who proposed the first question in the circular being absent, in the hope that he might yet arrive, it was agreed to make it the last in order; and the Convention therefore proceeded to the discussion of

Question 2. "*Can teeth generally be preserved, for a considerable length of time, after abscess has formed around the roots?*"

Dr. W. H. ATKINSON took the affirmative side of this question. He reported a case which occurred many years ago. Six pivot teeth were inserted, and, in a few days, the mouth was painful. He could not get the teeth out, and abscesses formed about the roots. Concluding to fall back on the principles of general surgery, he opened the abscesses, and applied creosote to the interior of the sacs. These soon healed, and twelve years afterward, these roots were still retained with comfort, and without return of the disease. That convinced him that if we could get a vent to an abscess, we could counteract and suppress it. For some time afterward, he set his pivot teeth with vents. He reported a great number and variety of cases of abscess successfully treated, but, from the rapidity of the report we are not able to give all.

Dr. SLOSSON corroborated Dr. A's views, that most cases of abscess can be cured, if the patient can be controlled, but that can not, in many cases, be done; for when a tooth is painful, the patient will generally have it out. He reported the case of a tooth, which, ten years ago, was affected with abscess, and quite loose. He made an opening through the gum and process to the end of the fang, and scraped off a rough surface of the process. The tooth is now healthy and useful. He had not carefully registered his cases, but successfully treated abscesses were of frequent occurrencs in his practice. The time is coming when this can all be controlled.

Dr. L. C. INGERSOLL remarked that a great difficulty with him in treating abscess, is, that he can't get his patients to come for the necessary treatment; and the reason is, they don't value their teeth. He had succeeded in some cases; but his experience in this department was not very extensive.

Dr. SLOSSON reported a case which came under his observation ten years ago. The front teeth had been roughly cut off. Severe inflammation, terminating in the formation of alveolar abscess, was the result. In this condition the patient applied to him. He treated the case a few weeks, and inserted pivot teeth. No return of abscess, unless very recently.

Dr. SPELMAN remarked that he had watched and recorded his cases with more attention since the last meeting of the Convention. About seven years ago, a young lady of delicate constitution had two teeth which he then considered too much diseased to fill. In December last she called to have other teeth filled, when he found the two referred to, with the crowns considerably broken, and with abscess at the roots. He resolved to treat them. He saw the patient two or three times a week. One of them has been filled for some time and seems healthy. The other is temporarily filled and does well thus far. In other cases he had not been so successful. He thought there was usually a little tenderness of the periosteum; and that, therefore, the nerve should always be preserved when it can be done. He thought he had used nitrate of silver too freely, but had now abandoned it. He uses, in its stead, creosote and tincture of iodine. The latter he regards as a good application in local inflammation. He would alternate the two. Decomposition, or change of properties, might result from mixing. He referred to the fact that tincture of iodine, creosote and water, form a colorless compound.

Dr. WATT had nothing new to offer. In general, he coincided with the remarks already offered. He referred to a case to corroborate the correctness of the principles of treatment already laid down. When the constitution is tolerably good, he would expect the treatment of alveolar abscess to be successful.

Adjourned till 7, P. M.

FIRST DAY—EVENING SESSION.

Question 3. "*Is it judicious to use arsenic, or any other powerful agent, to destroy the sensibility of dentine?*"

Dr. WATT remarked that he was on both sides of this question. He thought it was *not* judicious to use arsenic, merely to destroy sensibility of dentine; while he considered it highly so, in some cases, to use some "other powerful agent," for this purpose. He said that most of the agents used for the purpose referred to, act by virtue of their affinities for some one or more of the constituents of dentine, and, hence, may be called chemical remedies. And then the circumstances important to be noticed are the constituents likely to be acted on, the strength of the affinity, and the nature and properties of the compounds resulting from the action of the remedies. Creosote, tannin, chloride of zinc, nitrate of silver, and perhaps arsenic, act, principally, by virtue of their affinities for the aluminous, or gelatinous portion of the tooth. The active principle of creosote is carbolic acid, and a carbolate of albumen results from its action on alluminous tissues. Tannate of albumen results from the action of tannin, or tannic acid; and both these salts are insoluble in the fluids of the mouth, in water, or in an excess of albumen or either of the acids. The result of the combination is necessarily a loss of vitality. Accordingly, by the action of either of these, we have the sensitive portion of subjacent dentine covered by an elastic, insoluble, indestructible layer of dead matter, which exactly fits it, presenting no harsh or irregular surface, and excluding foreign substances, whether solid, liquid or gaseous. The insolubility of the compound is a guarantee against absorption of the remedy, and consequent danger to the pulp.

With arsenious acid the case is different. Either the arsenites of albumen, gelatine, etc., are poisonous, as well as soluble and liable to be absorbed, or they are readily decomposed, and the acid, becoming liberated, is ready to form new combinations, and thus the vitality of the pulp is endangered. All agree that the pulp may be reached by arsenic through a considerable thickness of dentine, yet there is some obscurity resting on the nature of its action on living tissues.

Chloride of zinc and nitrate of silver form salts with albumen, etc., differing in their chemical nature from the carbolate and tannate referred to, yet possessing nearly the same physical properties. The protecting layer formed by them is soluble in solutions of albumen, chloride of sodium, etc., yet insoluble in water and similar agents. The nature of the compounds prevent absorption; and, consequently, the pulp is not endangered by their use as it is with arsenious acid. He would use these, in some cases, and he considered them entitled to the appellation of "powerful" agents; yet he would not use arsenic, unless he was willing that the pulp should be destroyed.

Dr. Slosson had been in the habit of using cobalt for ten years, and thought, that with due discrimination, there was no risk. In some teeth it was not proper to use it. He had not used arsenic except as it exists in cobalt. After a patient is thirty years of age, he believed there was but little risk in the use of cobalt. In young persons he had witnessed soreness of the tooth after its use, but had not known of a case in which he had killed the nerve.

Dr. STRICKLAND had not been favorably impressed with the use of cabalt, in allaying sensibility. He had used arsenic in some cases, with perfect success. He had used it twenty years ago, in teeth which are good now. The first case in which he used it his patient was a young l ady. He applied a small quantity of it to her front teeth, and left it in all night. They were filled with comfort, and remained in ten years. They changed color, however, and he believed the pulps were dead. He got frightened, and abandoned its use. While he had chloride of zinc and creasote, he thought he would not return to arsenic, merely to allay sensibility of dentine. We can not tell, he said, how far the arsenic will penetrate, after its compounds are absorbed.

Dr. SLOSSON had lost teeth when he was satisfied that it resulted from filling on so sensitive a surface; but he had not, to his knowledge, lost any by the use of arsenic, as he used it in cobalt. If his experience corresponded with that reported by others, in the use of arsenic, he would abandon it too. He had even applied it to a nerve, and took off a part of it, without destroying the vitality of the tooth.

Dr. B. F. ROBINSON could not speak from experience on this article, as he never used either arsenic or cobalt to destroy sensibility of dentine.

Dr. W. B. INGERSOLL had used arsenic five or six years ago. A young lady, fifteen or sixteen years old, had small and very sensitive cavities in her front teeth. He applied a small quantity of arsenic, let it remain six hours, and filled the teeth next day. There was then no sensibility of dentine. In a week or ten days, the teeth became painful, and in two or three days afterwards he removed both fillings, and refilled them temporarily. In two or three weeks one of them was found to be quite red, as if injected with red blood. He drilled in and found the pulps dead. He filled them after the vitality was lost, and the red color gradually disappeared. He concluded to use arsenic no farther for this purpose.

Dr. MOORE remarked that he does not use arsenic—uses carbolic acid, which he makes himself, from white paper. He thought it as good as creosote in every respect, and better in some. Creosote he thought, injured the tooth, causing it to crumble like lime, especially if it touched the enamel.

Dr. W. I. ATKINSON said he had an unfortunate experiment in his own mouth. Used arsenic, and the tooth became very easy; but the peace was of short duration. Had the tooth taken out and split open. A thick stratum of dentine was found between the cavity and the pulp. At the part of the pulp next the plug, he found a small quantity of pus, with redness around it, the congestion extending to the apex.

He did not know but a nerve might absorb a little arsenic, and still live. However, he had discarded it, for the purpose under consideration. As to cobalt, he was aware of Dr. Slosson's success, and inquired if the success with it might not be explained by this theory of dose: the arsenic is so much diluted in the cobalt, that it might be all exhausted before reaching the central cavity, or, if not, the quantity taken up by the pulp will be extremely small. He was aware that it had an obtunding influence, and that part of the pulp might be removed, after its application without pain, and that in such cases it would bleed when cut. (To a question by Dr. Slosson.) It might bleed, and yet be dead. But creosote, he said, was his "darling remedy." If time would permit, he always used it. But if not, he would take chloride of zinc, in substance, and apply it for a minute or less, then apply creosote a minute or two, and proceed to excavate. The pain would be little felt.

Dr. DECAMP said he had tried chloride of zinc a few times, but he disliked the intense pain it produced. He had used cobalt, but had not tried arsenic for merely sensitive dentine. He succeeded best with creosote and tannin. He had known the sensibility return after the tooth was filled.

He referred to a case in which cobalt was used, and the sensibility was relieved. In two or three months the tooth was sensitive to heat and cold. He removed the filling and found the dentine very sensitive, used creosote and tannin, and filled. In two years it was again sensitive, but did not suffer from heat and cold. He took out the filling and found the dentine healthy—not at all sensitive. He treated variously for a month, then extracted and found the root affected with exostosis. He inquired if the treatment had anything to do in causing the exostosis? He thought it was often better to give time than to apply remedies for this condition.

Dr. STRICKLAND believed that arsenic is taken up by the dental tubuli and carried *wherever they go.* The depths of its effects will be in proportion to the time of its application. There *may* possibly be a change of color without total loss of vitality.

Dr. PERINE said that, in determining this question, we must take into consideration, the age and temperament of the patient, the texture of the tooth, and the time we have at our disposal. If he could control his patient, he would fill temporarily and wait till the sensibility abated. He had used, with good success, creosote, chloride of zinc, and kindred agents, in patients of mature age. He would endeavor to avoid their use with young patients.

Dr. C. F. INGERSOLL had never used arsenic for the purpose under consideration. He had used creosote and similar agents, but usually preferred a sharp instrument.

Dr. SLOSSON said he was in favor of temporary fillings and time, when practicable. He could not agree that cobalt acts as the President (Dr. Strickland) thinks arsenic does. If the vitality is destroyed, how does the sensibility return? He had seen many cases like that described by Dr. DeCamp.

Dr. STRICKLAND believed that the reaction set up was the cause of the sensibility, but did not believe in a final or full restoration to vitality.

Dr. TUTTLE thought that even for temporary filling it was necessary to excavate. He thought that to cut from centre to circumference, there was less pain than to cut otherwise. By observing this rule the use of remedies could often be dispensed with. He thought tin formed a good filling for a sensitive cavity, and suggested that the tin was oxydized by juices of the tooth.

Dr. W. H. ATKINSON remarked that the contents of the tubuli give vitallty to the dentine. Now if the instrument be dull, the walls of the tubuli are approximated when the cut is made, and their contents may press on the pulp. If this be true, the theory of Dr. Ingersoll is correct, and the sharp instrument is a good suggestion.

Dr. SPELMAN said, in reference to painful excavations, that when the decay is pared or dragged away, there is less pain than when the cut is made directly across the tubuli. Perhaps the fluid in the tubules is set in motion. This had been his theory, but he had not the impudence to state it before.

Question 4. "*What treatment will best preserve the vitality and health of exposed dental pulps?*"

Dr. SPELMAN said that for four months he had been following what was to him a new practice. When he finds the pulp visible through a thin layer, and exposed at a small point, he takes a little burnisher, its termination as nearly the shape of a flax-seed as possible, and presses up the dentine till he closes the opening, and then fills.

Dr. W. H. ATKINSON doubted the propriety of calling it pressing up the "dentine." Was it not cementum?

Dr. SPELMAN referred to eight successful cases treated in this way, and said that the idea was not original with him.

Adjourned till 8 A. M., to-morrow.

SECOND DAY—MORNING SESSION.

The discussion of Question 4, was resumed.

Dr. W. H. ATKINSON said he would treat with creosote and fill with, or without a cap, as the case may require.

When the pulp is exposed and healthy, he would apply either creosote or tannin, and then fill. His usual and favorite mode was to take a pledget of cotton on a blunt instrument, dip the cotton in creosote, and apply with pressure. There would be pain at first, but in a minute, or less, it would abate. He continues the pressure for a few minutes, and the pulp will be depressed so that we can fill with but little difficulty. He protects the exposed point with a horn cap, moistened with creosote, and fills as usual.

If the tooth aches, and the pulp is still covered with the animal portion of the tooth, he would again resort to creosote. He would use this article till he could find something better. He would not contend for riding a hobby "with head and tail up;" but when he rode, he wanted the animal to keep them up while able. After the tooth became easy, and he had removed all the softened dentine he considered necessary, he would apply the horn cap, as in complete exposure, pressing the cap in place with a pledget of cotton, where the atmospheric pressure would generally hold it. Then having a leaf of foil folded into a ribbon, and then into a proper sized layer, he would lay it over the cap, hold it in place with one instrument, while, with another, he would condense it, on opposing points prepared for its retention, and then complete the plug as usual.

The condition just described, he said, was congestion of the pulp ; but, if the disease pass beyond this, terminating in inflammation, then it will not, like the former, be relieved in five or six hours, but will require as many days. He had not much confidence in curative treatment of the pulp after suppuration had commenced. In the class of cases first described, he scarcely knew of a failure; in the latter, the failures would at least equal the successes.

Another important consideration in the treatment of exposed pulps, was the use of temporary fillings. He used tin for this purpose, in children's teeth. The success here, he thought, would be inversely as the age. He once removed a cement filling and gutta percha cap, from a tooth of a lady twenty years of age, and found the pulp still exposed and healthy. In adults he would plug permanently at the start, if the tooth were healthy,—in children he would not.

Dr. Strickland said that a good constitution was essential to successful treatment of exposed pulps. When the exposure is large, and the pulp unhealthy, he would prefer to destroy it. He had seen enough of capping to be satisfied that, in appropriate cases, the practice is judicious; but he occupied a middle ground. He preferred temporary fillings when he could watch the cases, and hoped to see many instances of ossific deposits protecting the pulp. When the exposure is small, and the tooth healthy, his treatment corresponds with that described by Dr. A. He referred to a case in which the pulp became covered by cementum, or osteo-dentine, in not more than six weeks. The exposure was small, and he applied creosote before inserting the temporary filling.

Dr. W. B. Ingersoll wished to know how soon we might expect a bony protection to be formed. He had known it to take place in a year or two. Others had witnessed it in much shorter time, and he wished to know the usual time required.

Dr. Strickland said it would be impossible to tell exactly. The age, health, and generel constitution of the patient would have a modifying effect.

Dr. Ambler described a case,—a lady eighteen years of age, with two exposed pulps. He treated with creosote and temporary fillings. In three months the bony protection was deposited, and the permanent plugs were inserted with complete success.

Dr. Tuttle said that when a nerve was wounded, he filled the cavity with dry cotton, to absorb the blood. When the hemorrhage ceased, he used a lead cap, and filled without applying any oscharetic. He sometimes used the horn cap, or tin, silver or gold. He had heard it suggested that tin and lead act as non-conductors. He prepared his cap by stamping through a plate with a properly shaped punch. The cap would thus have a concave and a convex surface. He then polished it and applied the concavity next the pulp. He did not use creosote—thought it must destroy a part of the pulp. He reported three cases in which a bony deposit had protected the pulp ; in other cases he had not been so successful. He remarked that the pulp recedes and bone is formed in the cavity, as age advances. He was convinced that creosote softens the bone, and has a tendency to destroy the pulp.

Dr. W. H. ATKINSON reported a case of large exposure. In three months removed the temporary filling, and found a considerable deposit of cementum, but the pulp was not quite protected. Dr. Butler, he said, could give a more definite account of the case.

Dr. BUTLER said that in the case referred to by Dr. A., the tooth became painful some three months after the insertion of the temporary filling. When this was removed there was no pus. While examining the case the pulp was accidentally wounded, and subsequently died in one fang. He concluded to destroy it with arsenic. The osseous deposit was extensive.

He once removed seven amalgam fillings from the mouth of a patient who said the pulps were exposed when they were inserted. There was no exposure when they were removed. (He did not approve of amalgam fillings in such cases.)

He described a case which he treated in the "Pennsylvania College," last winter. The pulp was exposed at a small point, he applied creosote, and, in the afternoon of the same day, filled it with crystal gold, using a horn cap. In three weeks after, the tooth had given no pain. His treatment of this tooth, he said, was in opposition to the teachings of all the professors there except one.

Dr. PERINE said he could not give the per cent. of successful cases; but if we could save one case we were making a mark. When the exposure was slight, he endeavored to make the treatment as mild and quick as possible. He capped with plaster of Paris, and had used the same as a protection to sensitive dentine. He had used creosote, tannin, etc., as described already by others. In answer to a question, he said he dries the plaster cap with warm air.

Dr. SLOSSON said he was glad those high in place now follow "our mode." The treatment of exposed pulps was his hobby eight or ten years ago. Now it appears that Dr. Butler has found that an amalgam plugger was in advance of him on this subject, and it seems he is ahead of the "Pennsylvania College." In closing, he said, that, though this is a hobby of his, yet he was careful not to put the gold cap on *wrong side up*. (Laughter.) He thought the nerve would often protect itself in a few days,—as soon as a wound on the finger, perhaps. He sometimes cuts off a portion of the nerve, applies creosote, and fills after the slough separates. He was in favor of temporary fillings, but a man might lose reputation by their use; and so he might by any honest course. He had seen many cases of osseous deposit. He used to destroy nerves, then tried to save them by partial excavation and a cap wrong side up, but now he cut off and treated as above described.

Dr. C. V. ATKINSON said, as to putting the cap wrong side up, his practice was to put the concave side next the pulp.

Dr. SLOSSON, (humorously.) That is what I meant by "wrong side up."

Dr. ATKINSON thought the stratum of air acted as a non-conductor.

Dr. SLOSSON thought, as the air expands and contracts, the pulp would be subject to pressure and consequent irritation.

Dr. DECAMP remarked that experience varies. He, however, generally put the cap on "wrong side up." He thought the air would be expelled. He said that he would leave softened dentine over the pulp, rather than expose it, and reported a case in which this softened dentine became hard. He now fills such cases temporarily, with the intention of refilling. He wants a very small space, the smaller the better, between the cap and the pulp.

Dr. LYMAN reported a case in which he accidentally exposed the pulp; (and was fearful the patient would expose him.) He applied dry cotton till the hemorrhage ceased, then applied nitrate of silver in substance, and sealed up with wax for three days. An eschar was formed, which he removed, and then re-applied the nitrate. In five days after, he again removed the eschar, sealed up with wax for a week, and then filled with gold. Six months after it was doing well.

Dr. SLOSSON said that he takes a copy of the cavity in wax, as a guide in making the cap.

Dr. PERINE wished to know the proportion of capped teeth saved.

Dr. C. V. ATKINSON thought about two thirds, in his practice.

Dr. SLOSSON had not kept a record, but the proportion was very large, three fourths perhaps.

Dr. DECAMP thought a large majority.

Dr. WAY thought that in filling from the bottom of the cavity, as he always does, there might be pressure on the pulp, in cases where it was protected only by a layer of softened dentine, as referred to a short time ago.

Dr. W. H. ATKINSON said, if we could tell the exact degree of vitality remaining in the dental tubuli, we could determine the proper course to pursue. Teeth with exposed pulps were once filled with a cement, and it was thought the oxyds had something to do with the bony deposit, but it would take place more readily under gold. He did not know why ossification would not take place at the termini as well as the bases of the tubuli.

Dr. WATT saw no reason why osseous matter should not be thrown out to protect the pulp, if foreign substances were excluded,—it would only be in accordance with what takes place in compound fracture. He was of the opinion that the pulp can deposit bony material only at the expense of its own bulk. Otherwise we might expect it, if properly protected, to throw out bony matter sufficient to fill the cavity of decay.

Dr. C. V. Atkinson had, several years ago, carefully examined some teeth which he thought confirmed the views just expressed in regard to the ossification of the pulp.

Question 5. *What preparation of gold, and what mode of inserting it, will most certainly and permanently arrest the progress of decay in teeth?*

Dr. Perine would only speak of the mode of using gold. He remarked that there are two divisions of gold foil, adhesive and non-adhesive; then we have crystal gold as well as foil. In filling a crown cavity, when no building out was required, he would use cylinders made of non-adhesive foil. To prepare these cylinders he would cut through the book with heavy shears, that the intervening paper might prevent the adhesion of the freshly cut edges. He then rolls the cylinders, by the usual modes, into the desired sizes and shapes, and anneals them in a platinum basin. In compound cavities he often used both adhesive and non-adhesive foil, and crystal gold, in the same cavity, as his judgment at the time dictated. He now used very small points, and filled but a few cavities in a day. He had sometimes used pallets. He spoke, by request, of his experiments with "soluble quartz," and exhibited specimens of teeth filled with his composition out of the mouth; and said he had fillings of the same material, in the mouth for the last six months, which are still doing well. When his experiments were matured he would give them to the profession. They would not be patented. The fillings just exhibited, he said, were made of precipitated quartz, mixed with powdered quartz and other substances. The materials are mixed in a mortar, and the mass hardens about as soon as an amalgam filling.

Dr. WATT remarked that as to the preparation of gold, the question was, perhaps, not very important. It was evident that good fillings can be made both from crystal gold and from foil. Quite a number of foil-makers are able to give us a good article; and but little difficulty need be experienced in obtaining pure gold foil, either adhesive or non-adhesive. To an inquiry as to the difference in the color of foils, from the same manufacturer, all professedly pure, he said the difference in color might arise from slight differences in manipulation, by which the particles became differently arranged. The color of pure gold, he said, varied with the circumstances under which it is placed. When precipitated in a granular form it is dark colored, and is rendered lighter by friction with a burnisher. Being neither purified nor contaminated by the burnisher, it was evident, he thought, that the change of color was due to a change of the arrangement of the ultimate particles.

Dr. W. H. ATKINSON thought that the color of foil was not modified by manipulation.

He thought the way to "most certainly and permanently arrest the progress of decay" was to fill with pure gold so as to make a solid mass that perfectly fills the cavity. He could now do all with adhesive foil that he could with crystal gold. He was first taught to use the rope; and he had gone through all the mutations and transformations, and was now back to the rope again, as his favorite mode. He believed he could make a stronger plug with adhesive foil than with crystal gold.

Dr. TUTTLE said he used various foils. He had seen the Utica foil, very adhesive at the start, lose its adhesiveness by keeping, like other varieties. His mode was to roll *any* foil into a rope, pass it through the flame of a spirit lamp, and clip into pellets, without touching it with his fingers. In a compound cavity he would make small pits, and fill them, then fasten a pellet from one filled pit to the other. Using finely serrated points, and condensing thoroughly as he proceeds, he would weld the filling into a solid mass. All the foils in market can be rendered adhesive by annealing.

Dr. W. B. INGERSOLL thought that much of the difference in foils was owing to manipulation ; but he thought, like Dr. Leslie, that pure gold, the world over, is about the same color.

Dr. STRICKLAND said, a gold beater had told him many years ago, that if foil were annealed over fine charcoal, a reddish tinge would be imparted to it.

It was, on motion of Dr. Perine, resolved that the remainder of the forenoon be devoted to the exhibition of specimens, instruments, etc.

SECOND DAY.—AFTERNOON SESSION.

After some time spent in miscellaneous remarks, mostly on peculiar traits of early practice and their results, the Convention proceeded to the discussion of the postponed

Question 1. *Under what circumstances is it* NOT *admissible to extract teeth?*

Dr. W. H. ATKINSON said, that as light advances, less and less extracting is done. He thought that in most cases, irregularity results from premature extraction of the primary teeth. Great care is required in this respect. Teeth affected with abscess ought not to be extracted, if one half the periosteum be alive. He thought teeth should not be extracted, merely because they are irregular, when the patient is only twenty-five years old.

Dr. MOORE gave it as his opinion that when the crown is destroyed by decay, the root of the temporary tooth will not be absorbed. He thought more than half the caries of the permanent teeth, results from neglect or bad treatment of the primary organs.

Dr. C. V. ATKINSON suggested that the decay of the primary set would directly affect the permanent, only as far back as the bicuspids, as the primary teeth extend no farther back than this. He was in favor of leaving the primary organs as long as possible.

Dr. PERINE would allow the primary organs to remain as long as possible. He would not be too easily frightened by irregularity as a little pressure, or even nature will generally make the necessary correction.

Dr. WATT being requested to give his views of the propriety of always extracting the primary teeth when affected by abscess, said that, if the tooth was causing but little irritation he would not extract; and even if there should be considerable inflammation, and he could control the patient, he would endeavor to reduce it, that the tooth might be retained, In answer to a question, he said that pregnancy, in itself considered, would not render it improper to extract an offending tooth. The pain of extraction, he considered less of a nervous shock, than one hour's severe tooth-ache.

Dr. DECAMP said he generally refuses to extract the primary teeth when called on by the parents. If they have remained till the permanent organs are making their appearance out of place, or if serious abscess appear, he would not hesitate to extract. He thought there was danger of the abscess injuring the germ of the permanent organ. He referred to the rapid growth which usually takes place at the age of puberty, and said that the expansion of the jaw was then frequently such, that teeth before crowded, had sufficient room, and fell into line.

Dr. PERINE reported a case of supposed cancer, caused by a portion of a deciduous root. This was removed, and the patient recovered.

Dr. SLOSSON thought that if a deciduous tooth was affected with abscess and very loose, it should be extracted; but if reasonably firm, and the irritation not great, it should be allowed to remain. The canines, he said, are often out of line. They should not be extracted. He had lost patients by refusing to extract, in such cases; but he still adhered to his position; the correctness of which he illustrated by a report of cases.

On motion of Dr. Perine, "Miscellaneous Business" was taken up.

Dr. PERINE stated that 53 oz., and 1 dwt. of platinum had been fused at a single melting recently in New York.

Dr. TUTTLE thought that remelted platinum was objectionable for practical purposes.

Dr. W. H. ATKINSON made some remarks on the importance of having a properly conducted dental depot in Northern Ohio. (Other members participated; but, as this is a local matter, we will not report it.—W.)

Dr. TUTTLE was heard at considerable length on mechanical dentistry. He described the advantages and disadvantages of "continuous gum," "hard rubber," and "cheoplastic" work. He exhibited a metal, (also specimens of it with teeth mounted on it,) used according to the "block tin" mode, which he said would not shrink in casting. This alloy is a a little darker in color than Blandy's; but has much the same appearance. Its composition. (if we heard correctly—W.) is

| | |
|---|---|
| Silver,................ | 8 pennyweights. |
| Tin,.................. | 40 " |
| Bismuth,.............. | 16 grains. |

He exhibited, also, the drawings of a pair of plate shears, for cutting the backings to any desired size and shape.

Dr. E. B. LOUD inquired if any of the members had tried the experiment of "extracting teeth by galvanism." He said the mode was to connect one pole of a battery with the forceps, and let the patient touch the other pole with his hand. At the moment connection is made, the tooth is to be taken out. The claim is that the pain is greatly lessened.

Dr. PERINE read a letter on local anasthesia, from Dr. C. W. Meble of Montreal, Canada, referring to an instrument of his own invention, for congealing the gums previous to extraction.

Adjourned till 7½, P. M.

SECOND DAY—EVENING SESSION.

The Convention met at 7½ o'clock.

On motion of Dr. W. H. Atkinson, it was resolved to take the initiatory steps to form a permanent society. On motion, Drs. Slosson, B. F. Robinson, W. B. Ingersoll, Ambler, J. A. Robinson, Strickland and Huntington were appointed a committee to draft a constitution and by-laws, and report at the next meeting of the Convention.

The remainder of the evening session was principally spent in discussing the merits and demerits of porcelain work, the discussions running nearly in the same channel, and conducted mainly by the same members as at the last meeting.

The Convention then adjourned, to meet at the same place, the first Tuesday of May, 1859, at 10 o'clock, A. M.

[After adjournment, the members residing in Cleveland entertained those from abroad, with "a competent portion of the good things of this life;" and the "outsiders" thanked the "insiders" for the abundant supply of the choice "insiders" of which they had just partaken. Verily, Cleveland is a good place to go to.—W.]

